# Mind the gap: Spatiotemporal patterns of airspace formation in duckweeds are regulated by hormones

**DOI:** 10.1093/plphys/kiae222

**Published:** 2024-04-18

**Authors:** Jiawen Chen

**Affiliations:** Assistant Features Editor, Plant Physiology, American Society of Plant Biologists; Division of Crop Biotechnics, Department of Biosystems, KU Leuven, 3001 Leuven, Belgium

The diversity of plant forms we see in nature is thanks to the adaptation of plant anatomy to many different environments and habitats. One of these adaptations is the aerenchyma, air spaces in plant leaves, shoots, and roots. These air spaces help facilitate gas conductivity, which is especially needed in waterlogged situations to facilitate oxygen transport from leaves to submerged roots. As such, aerenchyma formation can be a temporary adaptation to waterlogging in dryland species or a constitutive part of plant anatomy in wetland species (Evans 2003). As a temporary adaptation, other than hypoxia under water submergence, it can also be activated in response to other stresses such as nutrient deficiency and drought, and is precisely regulated through mechanisms such as ethylene signalling (Evans 2003).

The aerenchyma can form through two methods: lysigenous aerenchyma forms through programmed cell death (PCD), and schizogenous aerenchyma forms through differential growth of cells and cell separation (Evans 2003). Some species use only one of these, and others have both forms of aerenchyma. Aerenchyma formation in roots has been well studied, but the mechanisms behind leaf aerenchyma formation are less well understood. In leaves, aerenchyma formation occurs in three general steps: creating and expanding cavities, interconnecting those cavities, and linking with stomata for gas exchange.

Duckweeds (Lemnaceae family) are a great system to study aerenchyma formation, being an aquatic species that has well-developed aerenchyma in the fronds, which are vegetative tissues comparable to leaves. Duckweeds have experienced a revival as a model species in recent years due to increased genomic resources (Acosta et al. 2021). They are monocots that have a simple anatomical structure and commonly reproduce asexually through the budding off of clonal daughter fronds from the mother frond. The top of the frond (adaxial side) is exposed to air, while the bottom (abaxial side) is exposed to water, and only the adaxial side has stomata (Acosta et al. 2021, Fig. 1, A and B). The aerenchyma ensures gas transport between the adaxial and abaxial side of the frond and could also help with flotation.

**Figure 1.**
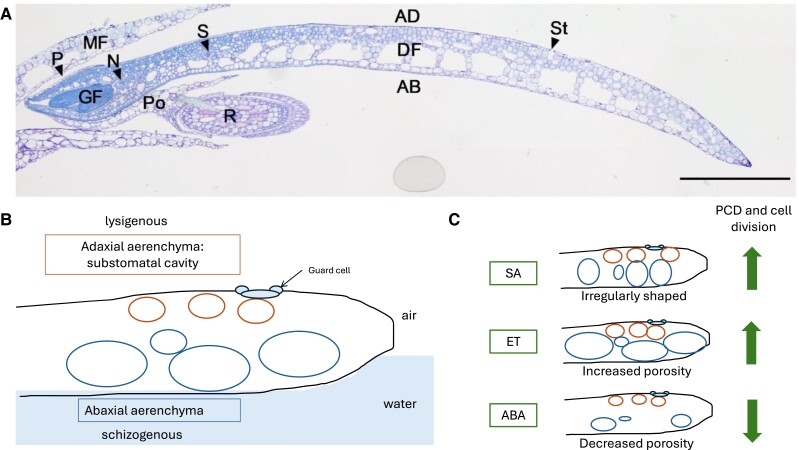
**Aerenchyma development of *S.polyrhiza* fronds is influenced by phytohormones. A)** Light microscopy transverse section of *S. polyrhiza* stage 6, stained with 0.01% (w/v) toluidine blue. Scale bar = 500 *µ*m. MF , mother frond; DF, daughter frond; GF, granddaughter frond; AD, adaxial parts of frond; AB, abaxial parts of frond; St, stomata; R, root; S, stem; N, node; P, prophyllyll; Po, pouch. Image taken from Kim et al. (2024). **B)** Schematic of aerenchyma anatomy in a developing *S. polyrhiza* frond. **C)** Summary of the effects of phytohormones (SA = salicylic acid, ET = ethylene, ABA = abscisic acid) on the pore formation patterns in aerenchyma anatomy and on processes of programmed cell death (PCD) for lysigenous aerenchyma and cell division for schizogenous aerenchyma formation.

In this issue of *Plant Physiology*, Kim et al. (2024) provide an overview of the spatiotemporal patterns of aerenchyma formation in developing fronds of the duckweed *Spirodela polyrhiza* by using light microscopy with histological staining and 3D imaging with x-ray micro-computed tomography (micro-CT). By performing RNA sequencing on different stages of frond development, they identified differential expression of phytohormone-related genes. Therefore, they looked at the effects of phytohormone treatment on aerenchyma formation and found specific developmental responses to abscisic acid (ABA), salicylic acid (SA), and 1-aminocyclopropanecarboxylic acid (ACC; as a proxy for ethylene).

The authors described eight developmental stages of frond formation in *S. polyrhiza* and observed that aerenchyma development occurred before frond volume expansion. Air spaces were visible first on the abaxial side and then on the adaxial side, where they would become substomatal cavities. Using micro-CT, the authors could closely look at connections between air spaces. They observed that the development of adaxial aerenchyma was faster than that of abaxial aerenchyma and that stage 3 was a crucial point at which interconnections between pores started forming, after which connections to the external environment created open pores. From stage 3 onward, substomatal cavities also formed through PCD. These results showed that *S. polyrhiza* formed both schizogenous aerenchyma during early frond development and lysigenous aerenchyma as substomatal cavities (Fig. 1B).

To understand the genes involved in the different stages of aerenchyma development, the authors performed RNA sequencing of fronds at stages 2 to 6. They found that PCD-related genes had stage-specific expression patterns that were opposite to those of cell division genes, confirming the processes observed through microscopy. Overall, they found clusters of both upregulated and downregulated genes, and a significantly upregulated group of genes included responses to phytohormones, mostly involved in later stages of frond development.

Exploring phytohormone regulation of aerenchyma formation more closely, the authors found that ACC and SA treatment had opposite effects to ABA treatment: ACC and SA caused increased PCD and cell division, whereas ABA decreased both. These hormones also affected the aerenchyma architecture, with ACC increasing porosity and ABA decreasing porosity. SA influenced the morphology of abaxial aerenchyma, and its effect was exacerbated by ACC, whereas ABA worked against the effects of SA. These results suggest an intricate network of phytohormonal regulation of the spatiotemporal patterns of aerenchyma development by finetuning cell division and cell death (Fig. 1C).

Kim et al. (2024) have created a detailed resource for *S. polyrhiza* frond development, with parallel microscopy and transcriptomics data detailing the stages of both schizogenous and lysigenous aerenchyma. The findings on the phytohormonal regulation of these processes in duckweed fronds expand on our knowledge of the well-studied effects of ethylene on lysigenous aerenchyma in roots of various crop species (Yamauchi and Nakazono 2022) and confirm the power of 3D micro-CT for studying intact structures like the aerenchyma in their native context (Jones et al. 2021). This study in duckweed also opens up avenues to explore aerenchyma in leaves of land plants to deepen our understanding on the diversification of aerenchyma formation.
